# Risk of Respiratory Infection following Diarrhea among Adult Women and Infants in Nepal

**DOI:** 10.4269/ajtmh.19-0405

**Published:** 2019-11-25

**Authors:** Kira L. Newman, Kathryn Gustafson, Janet A. Englund, Subarna K. Khatry, Stephen C. LeClerq, James M. Tielsch, Joanne Katz, Helen Y. Chu

**Affiliations:** 1 University of Washington, Seattle, Washington;; 2 Seattle Children’s Hospital, Seattle, Washington;; 3 Nepal Nutrition Intervention Project-Sarlahi (NNIPS), Sarlahi, Nepal;; 4 Johns Hopkins Bloomberg School of Public Health, Baltimore, Maryland;; 5 George Washington University Milken Institute School of Public Health, Washington, District of Columbia

## Abstract

Globally, diarrheal and respiratory infections are responsible for more than 24% of deaths in children aged less than 5 years. Historically, these disease entities have been studied separately; recent evidence suggests that preceding diarrheal disease may be a risk factor for subsequent respiratory illness. We used data from a community-based, prospective randomized trial of maternal influenza immunization of 3,693 pregnant women and their 3,646 infants conducted in rural Nepal from 2011 to 2014. A case-crossover design was used to determine whether the risk of respiratory infection in the 30 days following a diarrheal episode was increased compared with that 30 days prior. Diarrheal illness was a significant risk factor for subsequent respiratory illness in infants but not in women during pregnancy or in women up to six months postpartum. Diarrheal illness and respiratory infections remain important global sources of morbidity and mortality, and our study supports a causal relationship between them in infants.

Globally, diarrheal and respiratory infections are responsible for more than 24% of deaths in children aged less than 5 years and are major sources of adult morbidity and mortality.^1^ Decreasing childhood mortality is one of the major goals of public health.^2^ Historically, gastrointestinal and respiratory infections have been studied separately; however, evidence suggests that diarrheal disease may predispose infants and young children to respiratory illness and is associated with more severe illness.^3–6^ Furthermore, children who have concurrent diarrhea and respiratory illness are at a substantially increased risk of death compared with either illness alone.^4,7,8^ A proposed mechanism of this association is B and T cells cocirculation between the mucosal sites of the gut and the respiratory tract.^9–11^ Alterations in the gut microbiome through infection, medications, or other processes can alter mucosal immune responses and increase susceptibility to infection at other mucosal sites, including the lungs.^12^

To our knowledge, diarrhea as a risk factor for respiratory illness, particularly in adults, has not been well studied. The objective of this study was to assess the incidence of respiratory illness and infection following diarrheal illness among pregnant women, postpartum women, and infants in rural southern Nepal. This study is a secondary analysis of symptom data from two community-based, prospective randomized trials of maternal influenza immunization of pregnant women conducted in rural Nepal from 2011 to 2014.^13^ The trials prospectively enrolled 3,693 pregnant women and surveyed them weekly during pregnancy and up to 6 months postpartum for gastrointestinal and respiratory symptoms. Symptom data for their 3,646 infants were also collected weekly. Diarrheal illness episodes were defined as at least three watery bowel movements per day for one or more days,^14^ with seven or more diarrhea-free days between episodes. Respiratory illness is defined as the presence of fever with an additional respiratory symptom (i.e., cough, difficulty breathing, runny nose, or nasal congestion). For subjects with more than one episode of diarrhea, only the first episode was included in the analysis.

We conducted a case-crossover analysis in which each individual served as their own control to efficiently control for measured and unmeasured individual-level confounders.^15^ We compared the risk of respiratory infection in the 30 days immediately before an episode of diarrhea (control period) with the risk of respiratory infection in the 30 days immediately following the first day of a diarrhea episode (exposure period). We stratified the analysis by whether subjects were pregnant women, postpartum women, or infants. We then used conditional logistic regression to estimate the odds of respiratory infection in the control and exposure period. For all analyses, we used all available days and did not exclude individuals with incomplete follow-up time. Incidence of diarrhea was plotted over time and visually inspected for seasonality or other trends. We conducted a sensitivity analysis in which we examined a study period of 15 days pre- and post-diarrhea rather than 30 days. In addition, we performed post hoc power calculations.

All analyses were performed using R version 3.5.0 (R Foundation for Statistical Computing) in RStudio Version 1.1.453 (RStudio, Inc., Boston, MA). The Johns Hopkins University Bloomberg School of Public Health, Cincinnati Children’s Hospital, the Institute of Medicine at Tribhuvan University, and the Nepal Health Research Council Institutional Review Boards approved the trials on which this secondary analysis was based. The primary trial was registered with ClinicalTrials.gov (Trial #NCT01034254).

Of the 3,693 women enrolled in the trial, 525 (14.2%) had an episode of diarrhea reported during pregnancy and were included in our analysis and 226 (6.1%) had an episode of diarrhea reported during the postpartum period and were included. No women had diarrhea both during pregnancy and during the postpartum period. Of the 3,646 infants in the trial, 955 (26.2%) had an episode of diarrhea reported. There were no significant differences in the demographic or household characteristics of infants with diarrhea and without (Supplemental Table). Of pregnant women included, 344 (65.5%) had a full 60 days of follow-up during the combined exposure and control periods, as did 146 (64.6%) postpartum women and 737 (77.2%) infants. For the sensitivity analysis with 15 days exposure and control periods, 438 (83.4%) pregnant women had a full 30 days of follow-up during the combined exposure and control periods, as did 183 (81.0%) postpartum women and 878 (91.4%) infants. Table 1 summarizes characteristics of the subjects included in the case-crossover study.

**Table 1:** Characteristics of women and infants included in the study

Variable	Pregnant women	Postpartum women	Infants
	*N* = 525	*N* = 226	*N* = 955
Age (years for adults and days for infants), mean (SD)	23.5 (4.7)	24.2 (5.2)	90.8 (53.4)
Flu-vaccinated mother, *n* (%)	247 (47.1)	115 (51.1)	405 (50.3)
Nulliparous, *n* (%)	225 (43.1)	82 (36.4)	–
Preterm, *n* (%)	–	–	100 (10.5)
Low birth weight, *n* (%)	–	–	160 (20.5)
Small for gestational age, *n* (%)	–	–	275 (35.3)
Maternal smoking, *n* (%)	18 (3.6)	12 (5.7)	44 (4.9)
Maternal literacy, *n* (%)	300 (60.6)	124 (58.5)	570 (63.1)
Brahmin, *n* (%)	66 (12.9)	15 (6.8)	100 (10.7)
Madeshi, *n* (%)	202 (39.5)	84 (38.2)	293 (31.3)
Latrine, *n* (%)	240 (47.0)	87 (39.6)	455 (48.7)
Electricity, *n* (%)	466 (91.2)	197 (89.6)	841 (90.0)
Running water, *n* (%)	419 (82.0)	166 (75.8)	761 (81.5)
Indoor cookstove, *n* (%)	425 (83.0)	191 (86.4)	753 (80.5)
Household smoking, *n* (%)	221 (43.5)	119 (54.1)	429 (46.3)
People per room, mean (SD)	4.0 (2.7)	4.4 (3.5)	4.1 (3.2)
Children less than 5 years in household, *n* (%)	346 (65.9)	152 (67.3)	609 (63.8)
Respiratory episode in the exposure period, *n* (%)	27 (5.1)	5 (2.2)	185 (19.4)
Respiratory episode in the control period, *n* (%)	26 (5.0)	8 (3.5)	101 (10.6)

The incidence of respiratory episodes during the exposure and control periods (i.e., 60 days before and after the diarrheal episode) was approximately 2–5% in adults and 16–22% in infants. There was little evidence of seasonality. Preceding diarrhea was significantly associated with respiratory illness only among infants (odds ratio [OR]: 2.3, 95% CI: 1.73–3.1, Figure 1). Similarly, in the sensitivity analysis limiting the exposure and control periods to 15 days each, there was only a significant association between diarrhea and subsequent respiratory infection among infants (OR: 2.2, 95% CI: 1.5–3.1). In a post hoc power calculation based on an observed incidence of respiratory illness of 5%, this study had 80% power to detect a change from 5% incidence of respiratory illness to 9% incidence of respiratory illness, with approximately 500 of 3,700 subjects having diarrhea. Limiting the data to complete cases (344 pregnant women, 146 postpartum women, and 737 infants), we would estimate 30–60% power to detect a change from 5% incidence to 9% incidence in the adult women and 92% power among infants.

**Figure 1.**
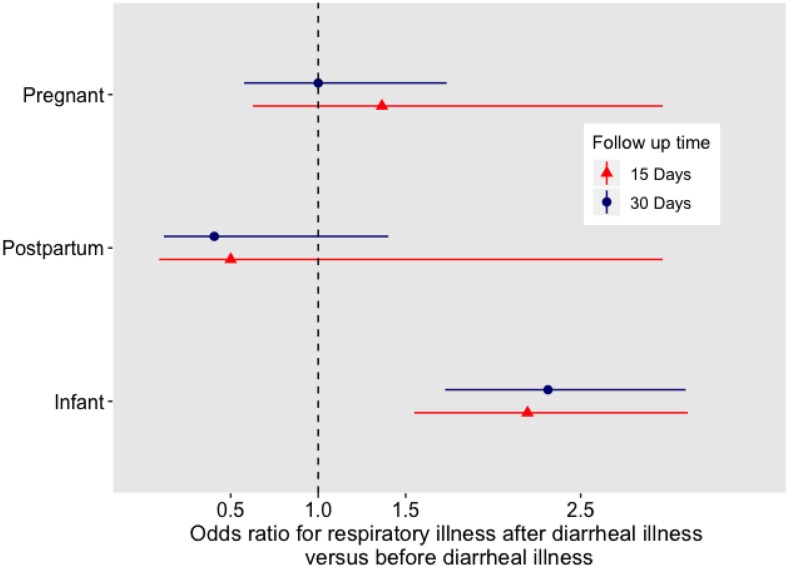
Odds ratios for likelihood of respiratory illness in the follow-up period following diarrheal illness episode compared with the period before diarrheal illness episode.

In this study of pregnant and postpartum women and their infants in Nepal, diarrheal illness was only a significant risk factor for subsequent respiratory illness among infants. Our findings are similar to those reported by Schmidt et al.,^5^ who reported an association between diarrhea and respiratory illness among children in Ghana. In the Ghanaian group, diarrhea within the preceding 2 weeks increased the risk of lower respiratory tract infection (hazard ratio 1.61). Similarly, data from cohorts of children in Nepal and India identified a correlation between diarrhea and respiratory infection that increases in strength as severity of illness increases.^6^ Our study investigated upper and lower respiratory illnesses together. This is different from prior work, which largely focused on pneumonia. A study of home visit data from Pakistan showed that diarrhea was a risk factor for pneumonia among children aged less than 5 years (hazard ratio 1.06) and that nearly a quarter of children with pneumonia had preceding diarrhea.^3^ Our study differs as well from earlier studies, which have looked at all children aged less than 5 years. Despite the fact that the infants in our study were largely breastfed during the study period, they still had relatively high incidence of diarrhea, but it may have been lower in our study than had we included children old enough to engage in complementary feeding, which other studies have found is associated with diarrhea.^16^

Limitations to our study include the lack of complete data in some subjects, some of which were lost to follow-up, potentially leading to bias; reliance on weekly recall for symptoms; and the low rate of respiratory illness among adults, leading to low power to detect all but a fairly substantial risk increase. However, this study is strengthened by its inclusion of pregnant and postpartum women in addition to infants.

In this study, we found that diarrhea was a risk factor for subsequent respiratory illness in infants but not among pregnant and postpartum women. However, in these groups of adults, we were underpowered to detect less than a near doubling of respiratory infection rate due to diarrhea. This study adds to the epidemiologic evidence regarding interactions between these infections.
